# Graphene oxide and its nanocomposites with EDTA or chitosan induce apoptosis in MCF-7 human breast cancer†

**DOI:** 10.1039/d1ra04345e

**Published:** 2021-08-31

**Authors:** Ahmed S. Doghish, Gharieb S. El-Sayyad, Al-Aliaa M. Sallam, Waleed F. Khalil, Waleed M. A. El Rouby

**Affiliations:** Department of Biochemistry, Faculty of Pharmacy (Boys), Al-Azhar University Nasr City Cairo 11651 Egypt; Drug Microbiology Lab, Drug Radiation Research Department, National Center for Radiation Research and Technology (NCRRT), Egyptian Atomic Energy Authority (EAEA) Cairo Egypt Gharieb.S.Elsayyad@eaea.org.eg; Chemical Engineering Department, Military Technical College (MTC) Egyptian Armed Forces Cairo Egypt; Biochemistry Department, Faculty of Pharmacy, Ain-Shams University Abassia Cairo 11566 Egypt; Department of Biochemistry, Faculty of Pharmacy, Badr University in Cairo (BUC) Badr City Cairo 11829 Egypt; Safety Fuel Cycle Department, Egyptian Nuclear and Radiological Regulatory Authority (ENRRA) Cairo Egypt; Material Science and Nanotechnology Department, Faculty of Postgraduate Studies for Advanced Sciences (PSAS), Beni-Suef University Beni-Suef 62511 Egypt waleedmohamedali@psas.bsu.edu.eg

## Abstract

To achieve the advanced anticancer activity of nanocomposites fabricated with graphene oxide (GO), a novel procedure was used during the fabrication of chitosan (CS) or ethylene diamine tetra acetic acid (EDTA). The synthesized GO-based nanocomposites were distinguished through different analytical techniques. The cytotoxic activity was examined using MTT assays against three different cancer cell lines. Cell cycle distribution and apoptosis were studied by flow cytometry. Caspase-8, caspase-9, and VEGFR-2 levels were determined using the ELISA technique. HRTEM results revealed a regular 2D thin sheet with a transparent surface in non-modified GO and for GO-CS, the surface of GO has clear cuts and lines had developed due to CS insertion. Concerning the MCF-7 breast cancer cell line, the lowest IC_50_ values were recorded, suggesting the most powerful cytotoxic effect on breast cancer cells. Treatment with GO-EDTA resulted in the lowest IC_50_ value of 3.8 ± 0.18 μg mL^−1^. As indicated by the annexin V-FITC apoptosis assay, the total apoptosis highest percentage was in GO-EDTA treatment (30.12%). In addition, the study of cell cycle analysis showed that GO-EDTA arrested the cell cycle primarily in the G0/G1 phase (33.74%). CS- and EDTA-conjugated GO showed an anti-cancer activity through their cytotoxic effect against the MCF-7 breast cancer cell line.

## Abstract 2

To achieve the advanced anticancer activity of nanocomposites fabricated with graphene oxide (GO), a novel procedure was used during the fabrication of chitosan (CS) or ethylene diamine tetra acetic acid (EDTA).

## Graphical abstract

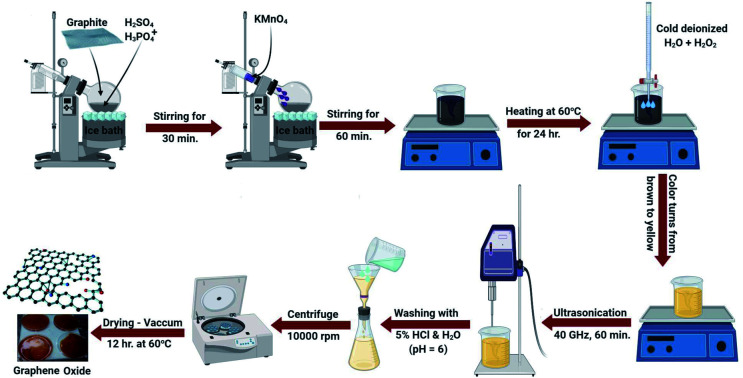

## Introduction

Cancer is considered the second leading cause of death in the world.^1^ Therefore, extensive and continuous research efforts are performed in this field.^2^ Clinically, each type of cancer requires a specific therapy that frequently encompasses one or more modalities, such as surgery, chemotherapy, and radiotherapy.^3^ However, these conservative cancer therapies have drawbacks limiting their therapeutic effectiveness. Moreover, traditional cancer treatments are still not enough to radically treat cancer or decrease the mortality rate. Therefore, finding more effective cancer therapy is required.^4^

In recent decades, the application of nanotechnology in cancer is an evolving field of research involving cooperation between different disciplines, including biology, chemistry, engineering, and medicine. The aim is to find novel technologies for more efficient cancer detection, diagnosis, and treatment.^5^ Using nanoparticles in cancer offers early detection as well as selective delivery of therapy to cancer cells not to normal cells by less invasive means.^1,3^ This is because nanoparticles can be designed to select tumor cells and slowly release the active anticancer agents there, both of which reduce systemic toxicity and improve the distribution and circulation time of these agents in the body.^2,4^

This smart strategy of tumor-selective targeting enlarges its application to embrace detection biosensors and phototherapy, in addition to theranostic lines. With that, various nano-carriers have previously been documented and supported by the food and drug administration agency (FDA) for cancer treatment and examination, implying the strength of cancer nanotechnology as the treatment of the future, promoting excellence and extending patients' lives.^6^

The smart strategy must use nanotechnology as a key point of smart nano-medicine for improving the functionality of medicine.^7,8^ Nanotechnology in large concerns the application of materials that maintain a very small size; commonly at the atomic or molecular order.^9–13^ It is commonly applied for biomedical goals and is a good technique for eliminating or minimizing the destructive potential of various pathogenic bacteria and fungi^1,14–16^ or functions as a powerful anti-cancer agent and a useful antioxidant factor.^12,17,18^ The important carrier characteristics of different nano-materials have maintained their goal in biomedical, agricultural, and environmental applications.^12,19–22^

GO possesses a great definite exterior area and special functional groups (epoxy, carboxyl, and hydroxyl groups) at its ends.^23^ It shows tremendous potential for the adsorption of different metal ions^23^ and unusual disinfectant potential upon remarkable pathogenic microorganisms^24,25^ and cancer cells.^6^ The outstanding surface area improves the capacity of GO application^26^ and so it may be utilized in viable biomedical and wastewater treatment application as a more reliable adsorbent to reduce various hazardous agents, cancer cells, and invading microbes.^27–29^ To improve the strength of GO in various treatments, some additional materials were assembled throughout GO and consequently used in various biomedical and environmental fields and within the examined materials such as CS and EDTA.^25,30,31^

EDTA is a cost-efficient chemical, usually-used as a chelating factor and applied in different purposes such as industrial laundry, in medical analysis as an anticoagulant agent, and for atomic power as a detergent.^32^ The antimicrobial and antitumor strength of EDTA due to the chelating means which develops through the cooperation with metal systems in microbial and cancer cells, which makes it an efficient center for microbial and cancer treatment.^2,33^ The design of the EDTA functional groups inside the outer surface of GO can basically advance the adsorption capacity and biomedical potential of GO sheets.^25,34,35^ It was found that GO-EDTA is an engaging adsorbent utilized for heavy metal and dangerous bacteria elimination. Where EDTA is joined with the pollutant substrate (heavy metal, bacteria and/or cancer cells), they perform a regular chelate between the hazardous materials.^25,34,35^

The natural polymer assembled with GO was CS, a substance originally obtained from chitin, a combination of carbohydrates collected from the outer structure of seafood such as shrimp and arthropods.^3,36^ CS was appointed as the natural adsorbent and inhibition substance to use with GO because it is common and has several advantages such as an amine group that is totally efficient between the metal ions, biodegradability, biocompatibility, and sustained application because of the non-poisonous operation.^37^

CS has obtained extensive applicability with other bio-polymers in general pharmaceutical devices as an inherent formulation excipient, which involve binding, separating, and tablet covering features.^38^ The polymer has also been studied as a potential adjuvant for swellable regulated drug delivery operations. The application of chitosan in unique drug delivery as muco-adhesive, gene, and peptide drug therapy through the oral route as well as its absorption improving impacts have been examined by several studies.^39^ CS shows multiple biological activities, particularly hypocholesterolemic, microbicide, and wound healing characteristics.^40^ Decreased toxicity linked with broad applicability presents it as a hopeful applicant not only as drug delivery, anticancer, or anti-inflammatory agents but also as a biologically active factor.^39,40^

In the current study, GO and its nanocomposite-based materials (GO-CS and GO-EDTA) were synthesized by a renewed Hummers method that maintains various benefits (it gives a system cleared and free of contaminants, is cost-effective, and presents a high yield). Accurate validations were performed to verify the anticancer characteristics of the integrated GO nano-composites based on their features like cleanliness, appearance and size, crystallinity, external charges, durability, and external morphology. Ultimately, possible anticancer activity determinations by cell viability assay, flow cytometric study for cell cycle and apoptosis exposure, and evaluation of caspase-8 and caspase-9 activities and VEGFR-2 were performed.

## Materials and methods

### Chemical and reagents

Paclitaxel (Taxol) was purchased from Sigma-Aldrich (St, Louis, MO, USA). 3-(4,5-Dimethyl-2-thiazolyl)-2,5-diphenyl-2*H*-tetrazolium bromide (MTT) and dimethyl sulfoxide (DMSO) were obtained from Sigma-Aldrich (Sigma, USA). Dulbecco's modified Eagle's medium (DMEM), fetal bovine serum (FBS), phosphate buffer saline (PBS), penicillin/streptomycin (Pen/Strep) solution (Pen/Strep), and trypsin-EDTA were purchased from Gibco (Gibco, TFS, Inc., USA). Additionally, chitosan was provided by Meron biopolymers company (India) with a degree of deacetylation of ≥85% and a medium molecular weight (190–200 kDa). The chemical structure, accurate degree, and the chemicals used in the GO synthetic methods are recorded in Table 1.

**Table 1:** Chemical structure, degree and supplier of the utilized chemicals

Reagents and chemicals	Chemical structure	Degree	Company
Sulphuric acid	H_2_SO_4_	98%	Sigma-Aldrich, UK
Phosphoric acid	H_3_PO_4_	85%	Sigma-Aldrich, UK
Hydrochloric acid	HCl	36.5%	Sigma-Aldrich, UK
Ethanol	C_2_H_5_OH	Analytical grade	Sigma-Aldrich, UK
Methanol	CH_3_OH	Analytical grade	Sigma-Aldrich, UK
Potassium permanganate	KMnO_4_	Analytical grade	WINLAB, UK
Sodium hydroxide	NaOH	Analytical grade	WINLAB, UK
Ethylene diamine tetraacetic acid (EDTA)	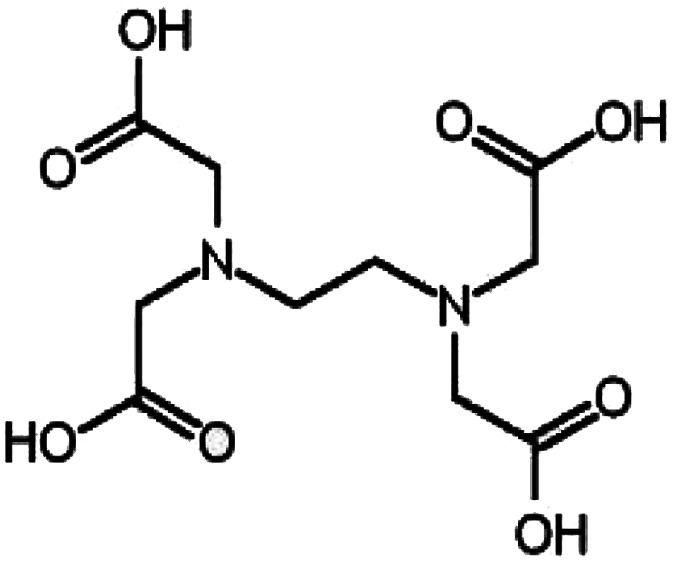	Analytical grade	WINLAB, UK
Chitosan (CS)	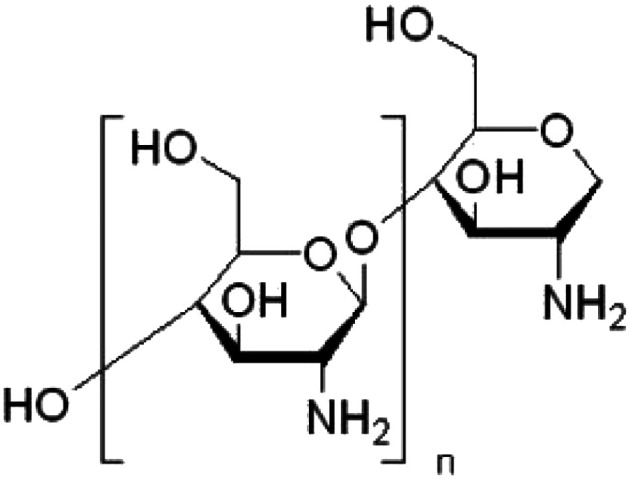	Degree of acetylation: more than 85%	Meron Biopolymers Company, India
Hydrogen peroxide	H_2_O_2_	30%	Alfa Company, India
Graphite fine powder	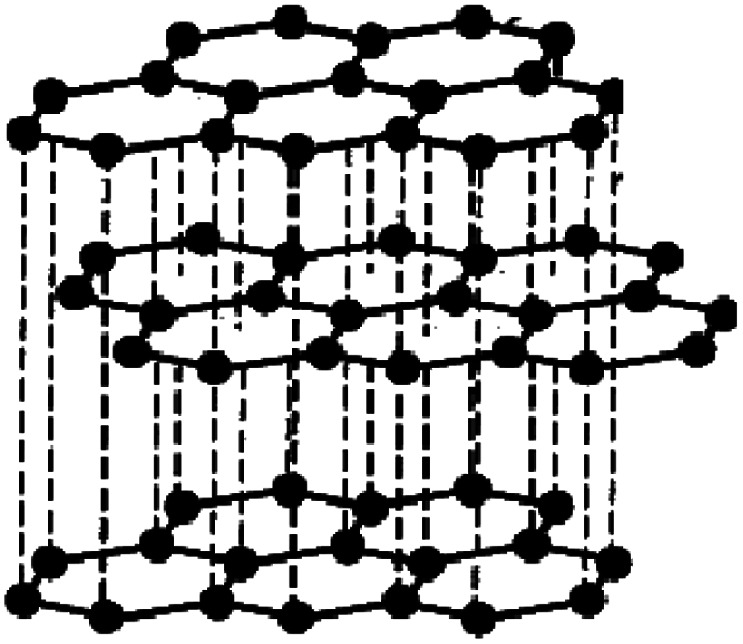	Particles size ˂50 μm	Merck Company, Germany

### Synthesis method

The process of chemical oxidation seems one of the current techniques utilized for GO layers synthesis from the starting graphite. This process includes graphite oxidation, peeling of the graphite oxide, and finally ultrasonication to extend the GO layers.

### Synthesis of graphene oxide

GO was developed by the improved Hummers method with some modification.^41^ The principal solution contained 60 mL phosphoric acid and 90 mL sulphuric acid combined in a 500 mL beaker in an ice bath (0 °C). 3.5 g of graphite powder was added to the solution with constant stirring (30 min). Next, potassium permanganate (20 g) was continuously added and the temperature was maintained at 0 °C. The resulting suspension was agitated for 60 min. The suspension was removed from the ice bath and kept at 60 °C on a hot plate for 1 day with constant stirring.

**Fig. 1:**
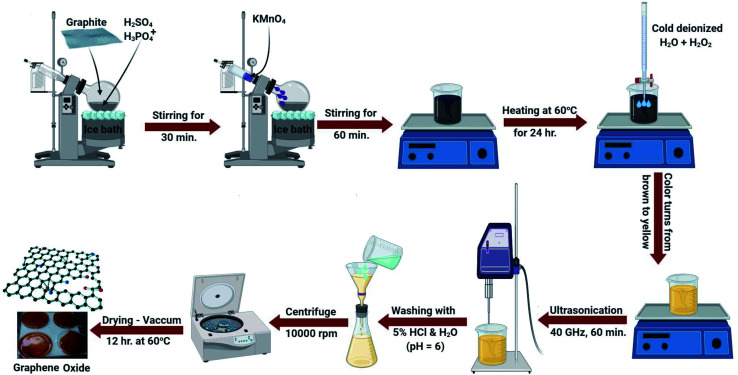
A schematic illustration for the preparation of GO.

The formed suspension was maintained at ambient temperature (25.0 ± 2 °C) and then 480 mL of frozen deionized water including 35 mL H_2_O_2_ solution was added. The solution color changed from hazy brown to dark yellow. Then, 1.5 L of distilled water was added and followed by ultrasonication for 1 hour. Lastly, the GO was obtained after centrifugation at 10 000 rpm and washing many times with distilled water and diluted HCl until a pH of 6 was attained. The generated GO was dried in a vacuum dryer at 60 °C for 12 h. The detailed process is explained in detail and schematically displayed in Fig. 1.

Certainly, in the initial Hummers design for GO synthesis, the reaction mixture solution such as H_2_SO_4_ : H_3_PO_4_ was 9 : 1, while a little alteration in this research is the difference in the ratio of acids. It was fixed as 90 mL H_2_SO_4_ and 60 mL H_3_PO_4_. Additionally, the quantity of KMnO_4_ was increased to 20 g. This change may be necessary for extended-range reproduction of GO where it increases the graphite oxidation.

### Fabrication of the GO-based EDTA nanocomposite

GO was modified with EDTA following the Jiyoung procedure.^42^ In brief, in a 1 L conical flask, about 0.80 g of the synthesized GO (Fig. 1) was added to 500 mL methanol and placed in a sonicator for 150 min. Next, 8.7 g of EDTA was added to the GO solution and left to agitate for about 17 h (at 65 °C) under reflux. After that, the reaction starts to end and about 250 mL methanol was added to eliminate the unreacted particles. The synthesized particles were isolated by centrifugation (15 000 rpm) for about 60 min accompanied by washing twice with double distilled water and finally methyl alcohol. The produced solution was dehydrated in a vacuum furnace for 13 h (at 65 °C). The final powder was called GO-EDTA and was dispersed in solution after moderate shaking.

### Fabrication of the GO-based CS nanocomposite

GO was fabricated with CS using the process defined by Yan Jiang *et al.*,^43^ with a small adjustment. 0.05 g of CS (with a degree of acetylation of ≥85% and a molar mass ranging from 190 to 200 kDa) was dissolved in 0.1% acetic acid. Following that, 0.50 g of the produced dehydrated GO (Fig. 1) was incorporated and sonicated with 250 mL distilled water for 120 min. Next, the adjusted GO solution was slowly added to the formed CS solution. The total solution was agitated for 60 min to obtain a permanent solution and ultimately, combined for 20 h to prepare the conjugated CS-GO solution. The generated GO-CS nano-powder was isolated by centrifugation after adjustment at 15 000 rpm for 65 min and washed with double distilled water many times to remove the acetic acid impurities. The synthesized nanocomposites were dried in a vacuum furnace for 15 h (65 °C).

For CS, viscosity performs an important part in the GO agglomeration. Furthermore, the concentration of GO cannot be ignored. Before beginning this research, the GO concentration was taken into consideration to decrease the agglomeration of GO. Also, the optimum concentration of CS solution (0.05%) was determined at which no GO agglomeration occurred.

### Validation of the synthesized materials

Several techniques were employed for the validation of the adapted materials. The X-ray diffraction (XRD) technique was conducted to determine the crystallinity and phases on a Brucker D8 Advance diffractometer using the radiation of Cu Kα by (*λ* = 1.540598 Å). The FT-Raman spectrum was reported using the BRUKER RFS 27 FT-Raman spectrometer, while FT-IR analysis was carried out by a JASCO FT-IR 3600 (400–4000 cm^−1^ wavenumber). About 5 to 10 mg fine powder of GO, GO-CS and GO-EDTA was placed on potassium bromide and ground until a homogeneous mixed powder was obtained. The mixed powders were pressed into a disc using a special mould and a hydraulic press. Then the FTIR spectra were recorded over the mid IR range (400–4000 cm^−1^). The principal appearance and size were determined by a high-resolution transmission electron microscope (HRTEM; JEM2100, JEOL, Japan). The indirect determination of the exterior charges of GO, GO-CS, and GO-EDTA was measured by the zeta potential analyzer, Malvern devise, UK. Finally, the surface shape, morphological characteristics, and behaviour of the synthesized GO-based nanocomposite were investigated by scanning electron microscopy (SEM; EVO-MA10, ZEISS).

### Cell lines

Human hepatic cancer (HepG2 and MH-22A), breast cancer (MCF-7) and normal human mammary epithelial (MCF-10A) cell lines were purchased from VACSERA (Giza, Egypt) and supplied through the American Type Culture Collection (ATCC; Manassas, USA). They were cultured in DMEM (Invitrogen/Life Technologies), supplemented with 10% FBS (Gibco, TFS Inc., USA) and 1% Pen/Strep solution (Gibco, TFS Inc., USA) at 37 °C in a 5% CO_2_ incubator.

### Cell viability assay

Cytotoxic activity was measured using the MTT assay (Sigma, USA).^44^ The cells were seeded at 1.2 × 10^4^ cells per well in 96-well plates and allowed to grow for 24 h. Media containing different concentrations of GO, GO-CS, and GO-EDTA were replaced after 24 hours. 100 μL of the MTT solution was applied to wells after 48 h (5 mg mL^−1^ in PBS) and maintained at 37 °C for 4 h. 100 μL of DMSO was applied to each well to dissolve the formazan crystals. The plates at 37 °C were incubated for 10 min. The optical density was quantified using a microplate reader (Epoc-2 C microplate reader, Bio Tek, USA) at 570 nm.

### Flow cytometric analysis for the cell cycle and apoptosis detection

The cell cycle in cultured cells for all groups was assesses by a cell cycle kit (Beckman Coulter, Inc., France, ref. C03552-AB) by using flow cytometry according to the manufacturer recommendations. Cell apoptosis in cultured cells was detected by annexin V-FITC apoptosis kit detection (BioVision, USA), with Catalog #: 101-25, for flow cytometry analysis (Beckman Coulter, Inc., USA), using the producer's standard protocols.^45^ Briefly, cells for all groups were cultured at 5 × 10^5^ cells per T75 flask and incubated overnight. After treatment with Taxol (8.24 μg mL^−1^), GO (35.8 μg mL^−1^), GO-CS (23.1 μg mL^−1^), and GO-EDTA (3.8 μg mL^−1^) or medium for 48 h, cells were collected and washed twice with PBS and stained with 5 μL annexin V-FITC and 5 μL PI in 1× binding buffer for 15 minutes at room temperature in the dark.

### Assessment of caspase-8 and caspase-9 activities and VEGFR-2

The assessment was carried out by using the caspase-8 (human) ELISA kit (EIA-4863) and the caspase-9 (human) ELISA kit (EIA-4860) (DRG International Inc., USA). While VEGFR-2 was done using the ELISA kit (Catalog #: OKAG02083) (AVIVA System Biology, USA) according to manufacturer instructions.

### Statistical analysis

GraphPad Prism 8.0 (software 2019, San Diego, CA, USA) was used to analyze all the results. All data were represented as means ± standard deviation from at least three independent experiments (*n* = 3). ANOVA and Tukey's multiple comparisons test were used to analyze the significant difference between all the groups' results. *P* < 0.05 was considered statistically significant.

## Results and discussion

### XRD analysis of the synthesized materials

**Fig. 2:**
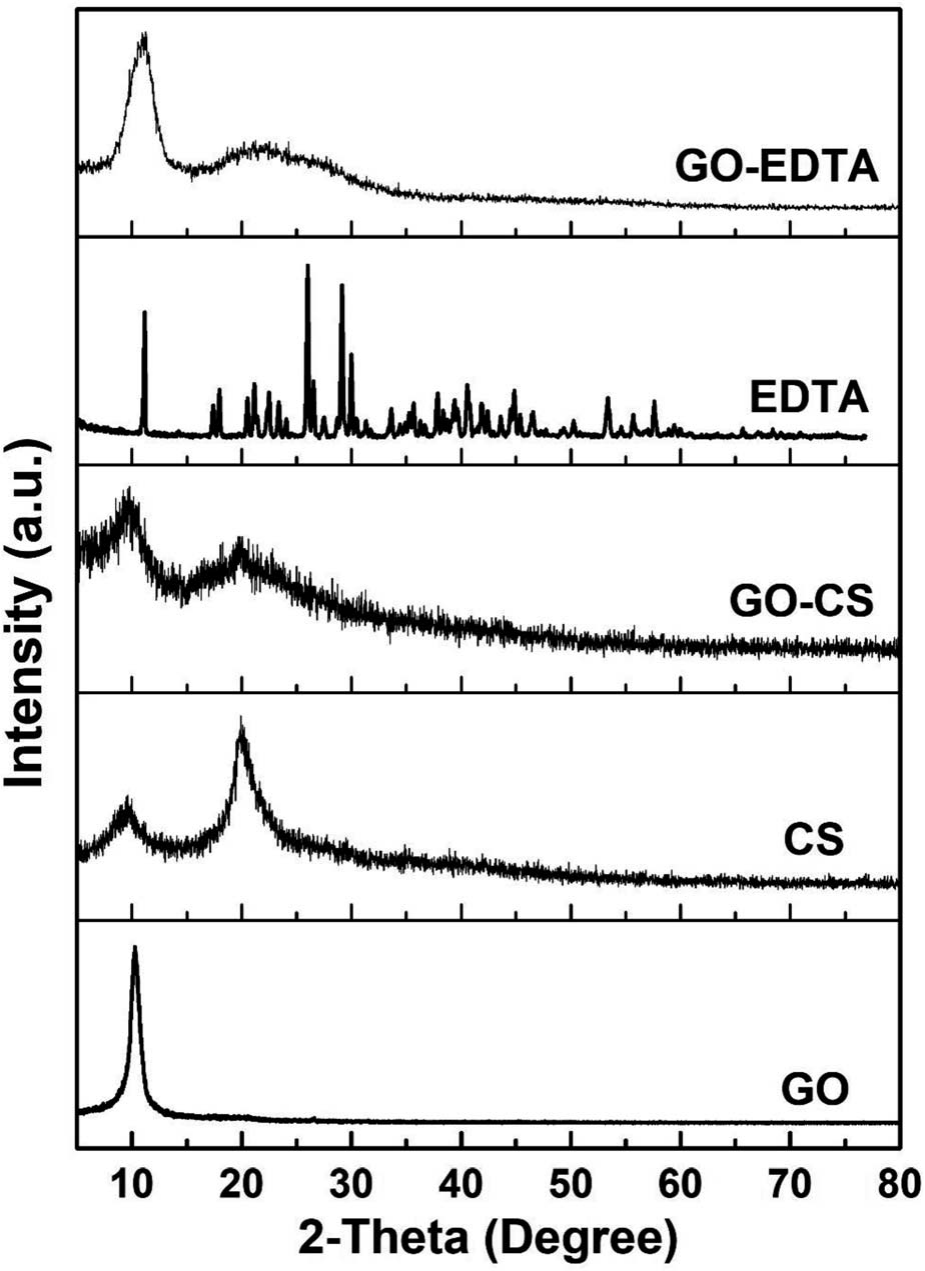
XRD patterns of GO, CS, EDTA, GO-CS and GO-EDTA.

XRD diffraction patterns of pure CS, pure EDTA, pure GO and its composites (GO-CS and GO-EDTA) are illustrated in Fig. 2. The characteristic peak of GO (2*θ* = 10.2°) clearly appeared as a sharp peak. This peak corresponds to the diffraction plane (002) of pure GO. The characteristic peaks of pure CS appeared at the 2*θ* value of 9.44° and 20.1°, confirming the crystalline nature of CS. When GO was modified with CS, from Fig. 2 it is clearly shown that a broad diffraction peak at 9.44° existed. This broad peak is attributed to the overlapping of the characteristic (002) peak of GO with the diffraction peak of CS.

From the XRD patterns of the GO modified CS nanocomposites, it is clearly seen that the intensity of the patterns was diminished, which is considered as an indication of the good distribution of the CS chains on the GO sheets. The XRD patterns of GO functionalized with EDTA show a clear peak related to GO while there are no peaks related to EDTA. The disappearance of the EDTA diffraction peaks is attributed to there being a small amount of EDTA linked to the GO surface. This amount of EDTA is less than the detection limit of XRD and cannot be detected, it is just functional groups on the GO surface. These mentioned results are in agreement with those previously reported in the literature.^46–50^

### Raman analysis of the GO containing samples

**Fig. 3:**
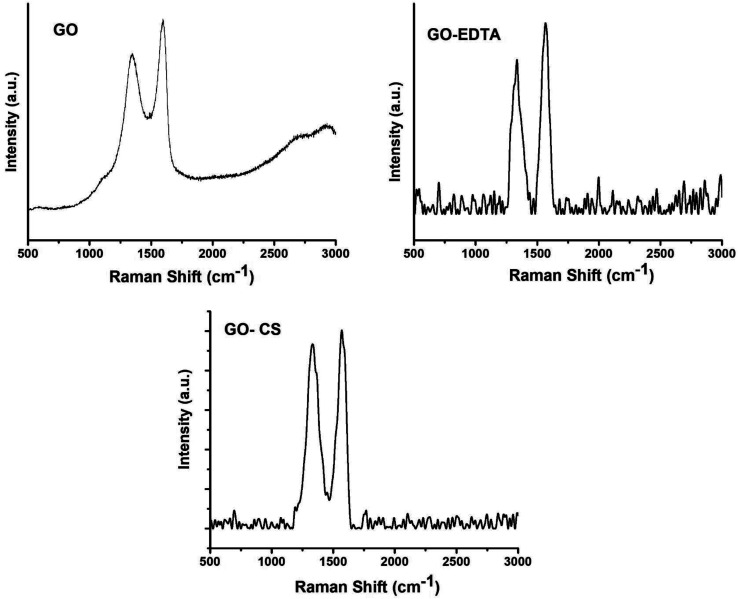
Raman spectra of GO, GO-CS and GO-EDTA samples.

As one of the best characterizing tools for carbon materials, Raman spectroscopy was used for characterizing the GO containing samples. Fig. 3 shows the recorded Raman spectra which exhibits two bands at 1350 and 1595 cm^−1^, corresponding to the D-band and G-band of GO, respectively. When the GO was modified with GO, the D-band and G-band shifted to 1332 and 1568 cm^−1^, respectively. In the case of the sample of GO functionalized with EDTA, the D-band and G-band were 1335 and 1567 cm^−1^, respectively.

As is well understood, the sp^2^ hybridized ordered carbon of the two-dimensional hexagon of graphite was expressed by the G-band. Simultaneously, the crack in the construction created by the sp^3^ hybridization disorder of carbon in the GO sheets was expressed by the D-band. To qualitatively describe the flaws and shifted ratios in the graphene framework, the intensity ratio (*I*_D_/*I*_G_) was determined from the Raman spectra of GO-containing samples. Based on the intensities of the D and G bands, it was decided that the *I*_D_/*I*_G_ value in GO modified with CS and GO functionalized with EDTA is higher than in the case of pure GO, meaning that the adjustment of GO with CS or EDTA enhances the breaks produced on the surface of GO.^51^

### Zeta potential measurements

The zeta potential measurements of GO containing samples were defined at pH 4, 6, 7.4 (physiological pH), and 9, as recorded in Table 2. From the calculated values, it can be assumed that the zeta potential effects show a negative impact by the sequence acquired by Salopek *et al.*^52^

**Table 2:** pH effect on the zeta potential of GO, GO-CS, and GO-EDTA

pH	Zeta potential (mV)		
	GO	GO-CS	GO-EDTA
4	−34.80	−30.46	−33.33
6	−39.14	−36.15	−46.25
7.4 (physiological pH)	−30.30	−35.78	−46.09
9	−24.66	−34.16	−45.87

The zeta potential of GO at pH ∼ 4 was found to be −34.80 mV, which is in line with the earlier reported data.^53^ When the GO surface was modified with CS, there was insignificant change in the zeta potential value when it was decreased to −30.46 mV. The decrease in the zeta potential value is due to the attached positively-charged CS on GO surface. At pH ∼ 4, the amino group in CS was protonated and induces a positive charge. This positive zeta potential of CS causes a decrease in the zeta potential value of GO-CS from −34.80 mV to −30.46 mV. The interaction between the positive and negative charge of CS and GO, respectively, confirm the strong interaction. The results were linked to a previous study, in which CS was charged on the surface of GO while the remaining charge was negative.^54^

### FTIR analysis of the pure and modified samples

**Fig. 4:**
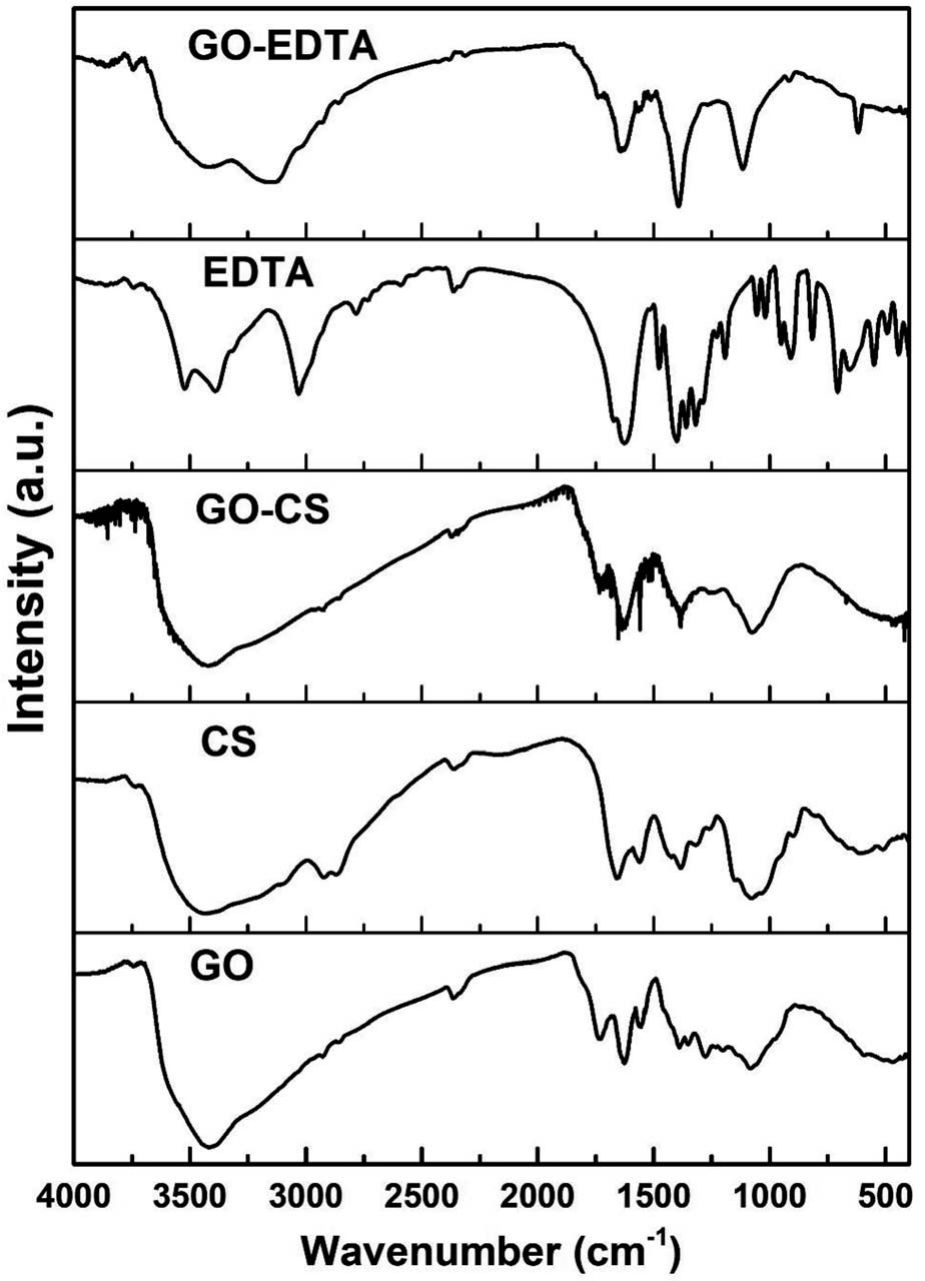
FTIR spectra of GO, CS, EDTA, GO-CS and GO-EDTA.

To distinguish the functional groups on the surface of pure and modified GO, the FTIR spectra were recorded for all samples and are illustrated in Fig. 4. In all the GO containing samples, the characteristic bands of the C–O stretching vibration of the carboxylic group are clearly seen at 1743 cm^−1^. In addition, the C–C stretching mode of the sp^2^ network appeared at 1639 cm^−1^. At 3429 cm^−1^, the stretching band of the OH group was observed in the case of samples containing GO. In the FTIR spectra of CS, there is a clear peak corresponding to the N–H stretching vibration of amino group located at 3437 cm^−1^. In addition, there is a peak located at 1636 cm^−1^ corresponding to the C=O stretching vibration of the amide group and another peak appeared at 1570 cm^−1^ which is related to the N–H bending of the –NH_2_ group.

From the FTIR spectra of the GO-CS specimen, it is evident that all the FTIR peaks of CS have developed in addition to different peaks placed at 1743 cm^−1^ due to the carboxyl groups on the GO surface. Due to interactions between CS and GO, these are altered in the GO-CS spectrum.

The recorded FTIR spectra of all the prepared GO containing samples are in agreement with those previously published in the literature.^48,55^ According to Shao *et al.*,^56^ due to the reaction within the epoxy functional groups described on the outside of GO and amino (NH_2_) groups near the CS surface, GO-CS composites are formed. They also mentioned that the GO-CS composite's system might be due to the presence of a covalent attachment made by the cross-linking of CS and GO, which limits the destruction of the amine parts in the CS.^56^

As is well-known from the literature^57,58^ and as confirmed from the present FTIR results, there is an interaction between the amino groups in the chitosan chains with the epoxy groups on the GO surface, resulting in the transformation of the primary amino (–NH_2_) group to secondary (–NH–) groups, as previously reported by Cao *et al.*^57^ In addition, CS contains amine and hydroxyl groups, while GO surfaces are fully with various functionalities. Thus, there is an interaction between these groups to form hydrogen bonds.^59^ Based on our previously published work;^25^ from the TGA measurements of pure CS there was about 45% weight loss of CS at a temperature range between 268 and 335 °C. While in case of GO-CS samples at the same temperature range (268 to 335 °C), the weight loss is about 8%. This means that the amount of CS that was loaded on the surface of GO is less than 4% of the total weight of the GO-CS sample.^25^

In terms of functionalizing GO with EDTA, the corresponding response was seen, with the detected peaks of pure EDTA appearing in the spectra of GO-EDTA plus the peaks of GO. The FTIR spectrum proved that the EDTA had strongly loaded on the GO surface.^34,42^ The FTIR spectra of all the checked samples were clearly in agreement with those previously reported in the literature.^48^

### Electron microscope examination

**Fig. 5:**
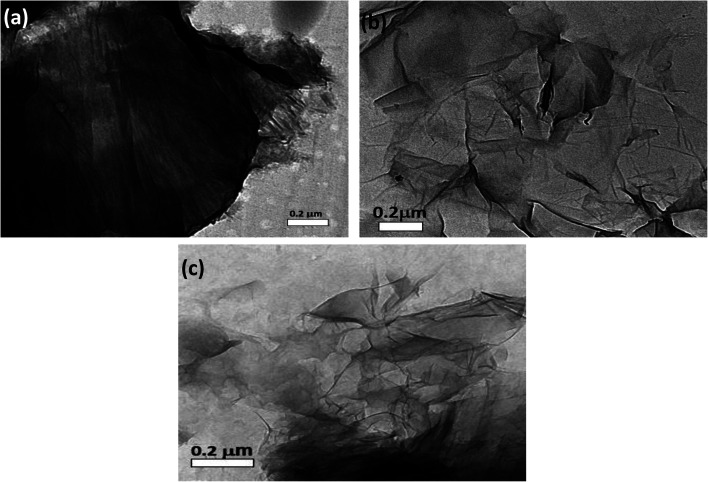
HRTEM images of (a) non-modified GO, (b) GO-CS, and (c) GO-EDTA.

High resolution transmission electron microscopy (HRTEM) was used to classify the unmodified GO layers, CS modified GO, and EDTA modified GO, as shown in Fig. 5a–c. In the case of non-modified GO, a standard 2D thin sheet with a smooth surface was obtained, as shown in Fig. 5a. This is attributable to the GO layers being heavily oxidized leading to carbon atom distortion in the GO sheets.

Fig. 5b shows an HRTEM image of GO altered by CS (GO-CS) composites, which reveals that the exterior surface of GO is cutted and lines have developed, which is due to the hydrogen-bonding synergy within GO and CS.^60^ The HRTEM representation of the GO sample following surface adjustment with EDTA (Fig. 5c) reveals that the layered construction of GO is furthermore entire and has not been damaged.

**Fig. 6:**
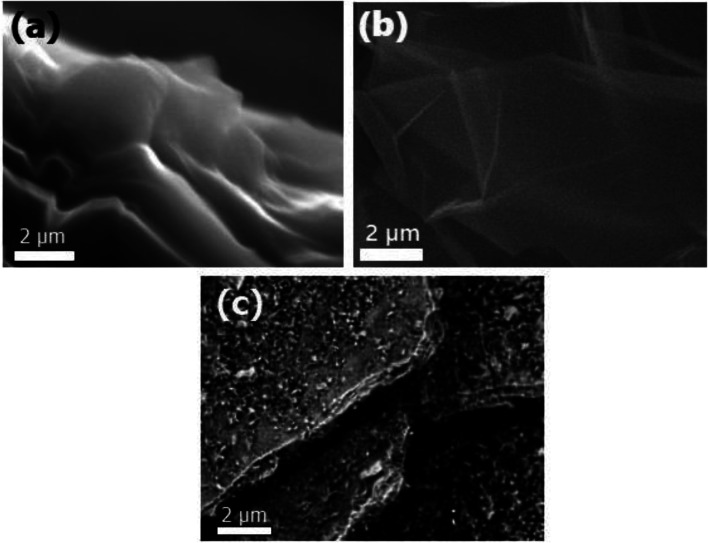
SEM images of pure GO (a), GO-CS (b), and GO-EDTA (c).

The SEM imaging was used to differentiate the surface and morphological highlights of the prepared GO, as well as the manufactured GO-CS and GO-EDTA, as presented in Fig. 6. Fig. 6a shows that the prepared GO's primary morphology has some fractures and holes, as well as an indeclinable internal structure. Furthermore, as shown in Fig. 6b, the assembled GO-CS nanocomposites are wholly refitted among the incorporated GO, with some distinguished edges and feathery lines, separate lines, and circle surface area, supporting the increase of CS over GO. Subsequently, SEM analysis of GO-EDTA (Fig. 6c) showed that some EDTA particles had connected with the edge of GO. This addition of GO, as well as CS and/or EDTA, indicates an increase in anticancer behavior.

### The cytotoxic effect of GO, GO-EDTA, and GO-CS against HepG2, MCF-7, MH-22A, and MCF-10A cell lines

**Fig. 7:**
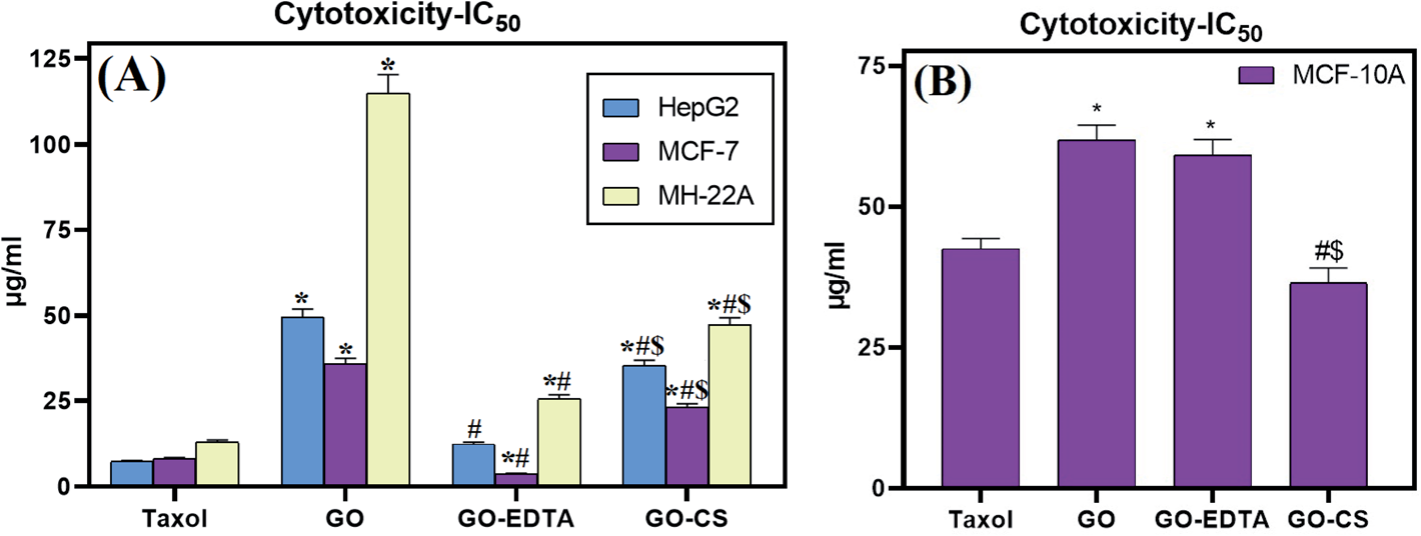
*In vitro* cytotoxic activities of Taxol, GO, GO-EDTA and GO-CS against (A) HepG2, MCF-7 and MH-22A cell lines and (B) the MCF-10A normal human mammary epithelial cell line. Data are represented as mean ± SD of three independent experiments. *Significant *P*-value from the Taxol group at *p* < 0.001, # significant *P*-value from GO group at *p* < 0.001, and $ significant *P*-value from GO-EDTA group at *p* < 0.001.

The cytotoxic effect of GO, GO-CS, and GO-EDTA was tested on three different tumor cell lines; HepG2 and MH-22A for hepatic cancer and MCF-7 for breast cancer. As illustrated in Fig. 7A, the lowest IC_50_ values were recorded on MCF-7, suggesting the most powerful cytotoxic effect being on breast cancer cells. Selective cytotoxicity was observed for these nanocomposites when tested on the normal human mammary epithelial cell line (MCF-10A) in comparison to the breast cancer cell line (MCF-7), as illustrated in Fig. 7B. On the one hand, for the control cells, treatment with GO-EDTA resulted in a IC_50_ value of 59.2 ± 2.8 μg mL^−1^. GO and GO-CS resulted in IC_50_ values of 61.9 ± 2.63 μg mL^−1^ and 36.5 ± 2.6 μg mL^−1^, respectively. However, the IC_50_ for Taxol was 42.5 ± 1.81 μg mL^−1^. On the other hand, for MCF-7 cells, treatment with GO-EDTA resulted in the lowest IC_50_ value of 3.8 ± 0.18 μg mL^−1^. Moreover, treatment with GO and GO-CS resulted in IC_50_ values of 35.8 ± 1.66 μg mL^−1^ and 23.1 ± 1.07 μg mL^−1^, respectively, while the IC_50_ for Taxol was 8.24 ± 0.38 μg mL^−1^.

This is the first study that evaluates this activity using this type of cells. Other studies using the same nanocomposites reported important antimicrobial activity against some pathogenic microbes.^25^ According to Isis *et al.*,^61^ no cytotoxicity was observed towards human corneal epithelial cell lines after 24 h exposure to 1000 μg mL^−1^ GO and GO-EDTA, suggesting that this nanomaterial has the potential for applications that have human exposure.

### The effect of GO, GO-EDTA, and GO-CS on apoptosis of MCF-7

**Fig. 8:**
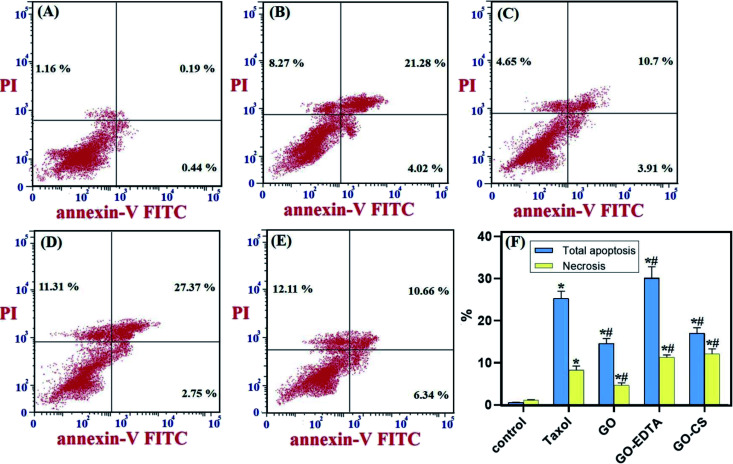
GO, GO-EDTA and GO-CS induce apoptosis in MCF-7 cells. MCF-7 cells were treated with Taxol (8.24 μg mL^−1^), GO (35.8 μg mL^−1^), GO-EDTA (3.8 μg mL^−1^), and GO-CS (23.1 μg mL^−1^) or medium for 48 h. (A) Control, (B) Taxol, (C) GO, (D) GO-EDTA and (E) GO-CS treated cells. (F) A graphical illustration of % of apoptotic and necrotic cells among different treated cells. * Significant *P*-value from control group at *p* < 0.01, # significant *P*-value from Taxol group at *p* < 0.01.

As depicted in Fig. 8, the distribution of cells in each quadrant was according to necrosis, late apoptosis, live cells, and early apoptosis (annexin V-positive cells). The percentage of total apoptosis was the highest in GO-EDTA treatment (30.12 ± 2.71%). For GO, GO-CS, and Taxol, the percentages were 14.6 ± 1.16%, 17 ± 1.36%, and 25.3 ± 1.73%, respectively, while in the control cells, the total apoptosis was 1.79 ± 0.02%.

Moreover, for the necrotic cells percentage, it was dramatically high in GO-EDTA treatment (11.31 ± 0.65%). For GO, GO-CS, and Taxol treated cells, it was 4.65 ± 0.604%, 12.11 ± 1.2%, and 8.27 ± 0.99%, respectively. Control cells showed only 1.16 ± 0.09% necrosis. These findings highlight that the anticancer activity of these nanocomposites is owing to their induction of both apoptosis and necrosis of cells.

EDTA-nanocomposites could improve some anticancer drugs solubility and bioavailability for cancer cells. For example, it was proved that conjugation of camptothecin with β-cyclodextrin and iron oxide NPs (Fe_3_O_4_ NPs) cross-linked with EDTA achieved improved camptothecin efficiency as an anti-cancer drug in colon cancer. Also, it reduces anticancer drug toxicity.^62^

### Cell cycle analysis

**Fig. 9:**
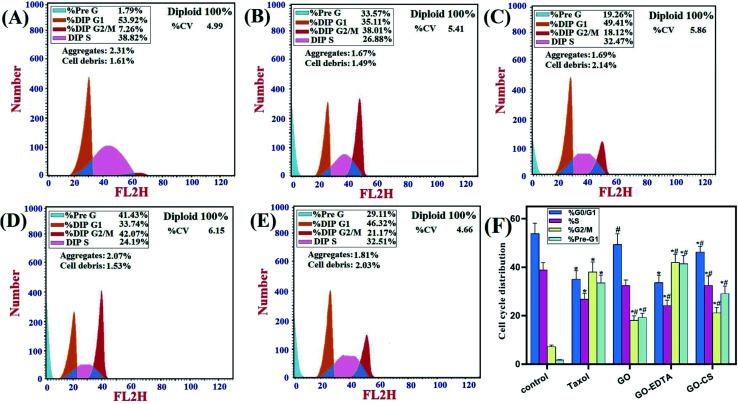
Flow cytometry analysis for cell cycle distribution of MCF-7 cells. MCF-7 cells were treated with Taxol (8.24 μg mL^−1^), GO (35.8 μg mL^−1^), GO-EDTA (3.8 μg mL^−1^), and GO-CS (23.1 μg mL^−1^) or medium for 48 h. (A) Control, (B) Taxol, (C) GO, (D) GO-EDTA and (E) GO-CS treated cells. (F) A graphical illustration of cell cycle distribution analysis among different treated cells. * Significant *P*-value from control group at *p* < 0.01, # significant *P*-value from Taxol group at *p* < 0.01.

Fig. 9 represents the cell cycle distribution of MCF-7 cells, treatment with GO-EDTA showed a significant increase in apoptosis by increasing cells in the pre-G1 phase (41.43 ± 3.4%). For GO, GO-CS, and Taxol, the percentages were 19.26 ± 1.76%, 29.11 ± 3.2%, and 33.57 ± 3.22%, respectively, compared to 1.79 ± 0.13% in control cells. Interestingly, treatment with GO-EDTA showed a significant decrease in cells arrested in the G1/G0 phase of the cell cycle (33.74 ± 2.99%). For GO, GO-CS, and Taxol, the percentages were 49.41 ± 4.44%, 46.32 ± 2.316%, and 35.11 ± 3.51%, respectively, compared to 53.92 ± 4.31% in control cells.

Moreover, GO-EDTA treatment produced a significant decrease in cells in the S phase (24.19 ± 2.23%). For GO, GO-CS, and Taxol, the percentages were 32.47 ± 2.28%, 32.51 ± 3.9%, and 26.88 ± 2.42%, respectively, compared to 38.82 ± 3.1% in control cells. On the other hand, GO-EDTA treatment resulted in a significant increase in cells accumulated in the G2/M phase of the cell cycle (42.07 ± 3.31%). On the other hand, the increase was significantly less in GO, GO-CS, and Taxol at 18.12 ± 1.84%, 21.17 ± 2.21%, and 38.01 ± 4.21%, correspondingly. In comparison, 7.26 ± 0.65% of control cells were present in the G2/M phase.

These results demonstrate a change in the cell cycle dynamics in response to nanocomposite treatment with GO-EDTA particles showing the highest efficiency. The arrest at the G2/M checkpoint indicates survival with DNA damage, which may activate either repair or apoptosis-like programs. Additionally, the increase in cells arrested in the pre-G1 phase supports the shift towards apoptosis.

### The effect of GO, GO-EDTA, and GO-CS on caspase 8 and caspase 9 activities

**Fig. 10:**
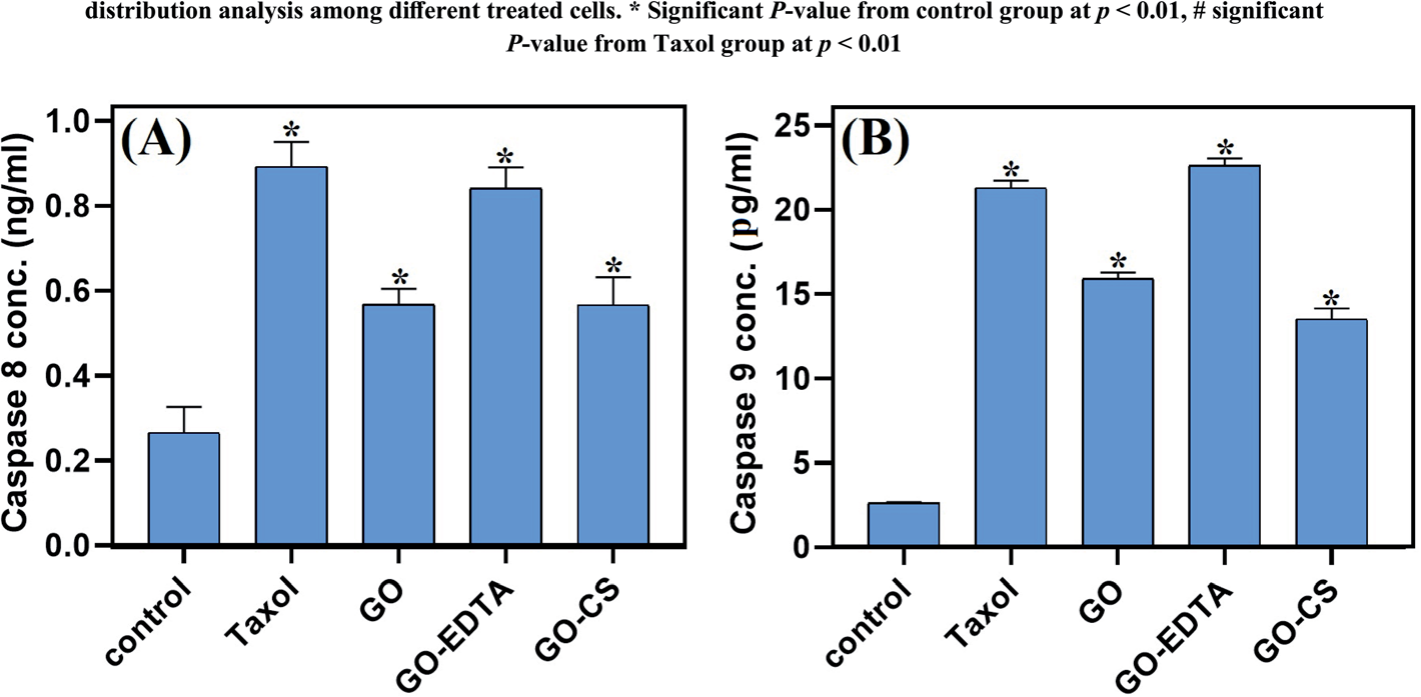
Effects of GO, GO-EDTA and GO-CS on the caspase-8 and caspase-9 activity (A and B, respectively) in MCF-7 cells compared to Taxol. MCF-7 cells were treated with Taxol (8.24 μg mL^−1^), GO (35.8 μg mL^−1^), GO-EDTA (3.8 μg mL^−1^), and GO-CS (23.1 μg mL^−1^) or medium for 48 h. Data are represented as mean ± SD, *: significant from control group at *P* value < 0.0001.

The effect of GO, GO-EDTA, and GO-CS on the apoptosis markers caspase-8 and -9 is described in Fig. 10. The activity of caspase 8 and caspase 9 was significantly increased with treatment of MCF-7 cells with GO (0.568 ± 0.037 ng mL^−1^ and 15.9 ± 0.38 pg mL^−1^; respectively) and with GO-CS (0.566 ± 0.066 ng mL^−1^ and 13.5 ± 0.65 pg mL^−1^; respectively) in comparison to the control (0.266 ± 0.061 ng mL^−1^ and 2.614 ± 0.07 pg mL^−1^; respectively). Moreover, treatment with GO-EDTA achieved the highest elevation in both caspase-8 and -9 activities (0.842 ± 0.049 ng mL^−1^ and 22.63 ± 0.42 pg mL^−1^; respectively) when compared with either GO or GO-CS treatments. Activation of caspases such as caspase-8 and caspase-9 is responsible for apoptosis, resulting in activation of some DNA fragmentation enzymes.^63^ Increased caspase-8 and -9 activities further strengthens the nanocomposite induced apoptosis in MCF-7 cells *via* caspase 8 and caspase 9 dependent manner. According to El-Naggar and El-Said,^64^ EDTA alone had no antitumor effect and did not alter the cisplatin anti-tumor efficacy. Furthermore, the application of EDTA in drug delivery systems could significantly reduce drug toxicity without influencing antitumor activity, as previously reported by Song *et al.*^65^

### The effect of GO, GO-EDTA, and GO-CS on VEGFR-2

**Fig. 11:**
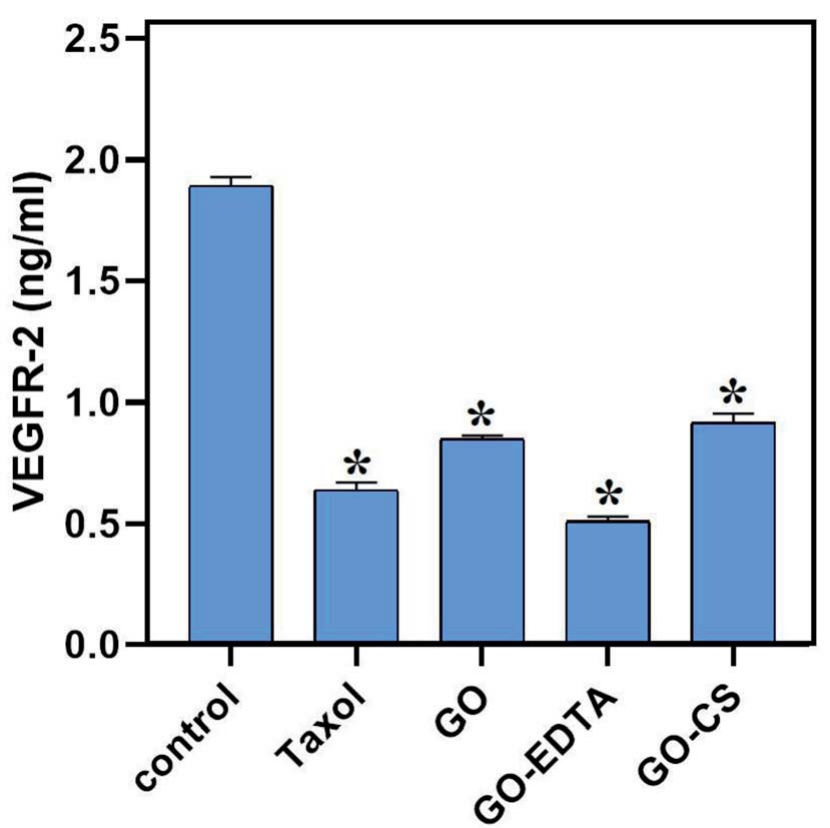
Effects of GO, GO-EDTA and GO-CS on VEGFR-2 in MCF-7 cells compared to Taxol. MCF-7 cells were treated with Taxol (8.24 μg mL^−1^), GO (35.8 μg mL^−1^), GO-EDTA (3.8 μg mL^−1^), and GO-CS (23.1 μg mL^−1^) or medium for 48 h. Data are represented as mean ± SD, *: significant from control group at *P* value < 0.0001.

Controversially, as represented in Fig. 11, our nanocomposites significantly decreased the level of VEGFR-2 compared to the control (1.893 ± 0.036 ng mL^−1^), where GO-EDTA treatment represented the lowest levels of VEGFR-2 (0.511 ± 0.017 ng mL^−1^) with regard to GO (0.848 ± 0.015 ng mL^−1^) and GO-CS (0.916 ± 0.037 ng mL^−1^). This result was in accordance with Falcon *et al.*,^66^ who stated that excessive activation of VEGFR-2 was found to mediate angiogenesis that promotes solid tumor growth and inhibits the VEGFR-2 pathway, which has become a key therapy for many cancer types, including breast cancer.^66^

## Conclusions

The impact of CS and EDTA on the crystal structure of treated GO was identified by XRD analysis. The basic form and size were determined by HR-TEM, which revealed that GO was a standard 2D thin layer with a uniform surface. In GO-CS, the exterior surface of GO was clearly cut and lines had formed, which was attributed to the hydrogen bonding interaction of GO with CS. On the other hand, for the GO sample formed following surface adjustment with EDTA, the image reveals that the layered construction of GO still maintained its structure without any defects. Concerning the MCF-7 breast cancer cell line, the lowest IC_50_ values were reported, indicating the most powerful cytotoxic effect being on breast cancer cells. Treatment with GO-EDTA occurred with the lowest IC_50_ value of 3.8 ± 0.18 μg mL^−1^. As indicated by the annexin V-FITC apoptosis assay, the highest percentage of total apoptosis was in the GO-EDTA treatment (30.12%). For GO, GO-CS, and Taxol, the percentages were 14.6%, 17.0%, and 25.3%, respectively. Also, the study of our outcomes by cell cycle analysis showed that GO-EDTA arrested the cell cycle primarily in the G0/G1 phase (33.74%). This result was supported by the highest elevation in both caspase-8 and -9 activities (0.842 ± 0.049 ng mL^−1^ and 22.63 ± 0.42 pg mL^−1^; respectively) in the GO-EDTA group when compared with either the GO or GO-CS groups, where GO-EDTA treatment represented the lowest levels of VEGFR-2 (0.511 ± 0.017 ng mL^−1^) with regard to GO (0.848 ± 0.015 ng mL^−1^) and GO-CS (0.916 ± 0.037 ng mL^−1^).

## Author contributions

**Ahmed S. Doghish:** conceptualization, data curation, formal analysis, investigation, methodology, resources, software, validation, writing – original draft. **Gharieb S. El-Sayyad:** conceptualization, data curation, formal analysis, investigation, methodology, resources, software, validation, visualization, writing – original draft, writing – review & editing. **Al-Aliaa M. Sallam:** conceptualization, data curation, formal analysis, investigation, methodology, resources, software, validation, writing – original draft. **Waleed F. Khalil:** conceptualization, data curation, formal analysis, investigation, methodology, resources, software, validation, visualization, writing – original draft, writing – review & editing. **Waleed M. A. El Rouby:** conceptualization, data curation, formal analysis, investigation, methodology, resources, software, validation, visualization, writing – original draft, writing – review & editing.

## Conflicts of interest

The authors declare that they have no conflict of interest.

## Supplementary Material

RA-011-D1RA04345E-s001
