# Supplementary material for: Graphene oxide and its nanocomposites with EDTA or chitosan induce apoptosis in MCF-7 human breast cancer
Source: RSC Adv. 2021 Aug 31;11(46):29052–64. doi: 10.1039/d1ra04345e (PMC9038105; doi:10.1039/d1ra04345e)
Supplement: RA-011-D1RA04345E-s001 [file RA-011-D1RA04345E-s001.pdf]

Electronic Supplementary Material (ESI) for RSC Advances.
This journal is © The Royal Society of Chemistry 2021

Researcher : Dr.Ahmed Soliman email: ahmed_doghish@azhar.edu.eg
Assay : Cell Cycle Analysis
Samples : 05 *3 samples
cell line : ---
Ref. : ---
Date : 23-09-2020
Reader : BD FACSCalibur
Kit used : Propidium Iodide Flow Cytometry Kit
Solvent :
Assay samples : Cell culture

Lab Report

**ser**	Sample data	Results DNA content											
	**code**	%G0-G1			%S			%G2/M			%Pre-G1		
1	Cont. MCF7	57.40	49.10	55.25	42.39	37.26	36.80	7.60	6.51	7.68	1.91	1.66	1.78
2	Taxol/MCF7	38.76	31.77	34.80	29.47	26.49	24.67	41.77	38.8	33.47	37.10	32.85	30.77
3	GO/MCF7	54.53	47.00	46.69	34.44	33.00	29.96	19.70	18.57	16.10	19.87	20.63	17.27
4	GO-EDTA/MCF7	36.97	31.05	33.20	26.70	23.38	22.48	45.57	41.66	38.99	41.19	44.94	38.15
5	GO-CS/MCF7	48.74	46.10	44.11	36.93	31.08	29.53	23.42	21.09	19.00	26.00	32.44	28.90

	%G0/G1		%S		%G2/M		%Pre-G1	
	Mean	SD	Mean	SD	Mean	SD	Mean	SD
control	53.92	4.31	38.82	3.1	7.26	0.65	1.79	0.13
Taxol	35.11	3.51	26.88	2.42	38.01	4.21	33.57	3.22
GO	49.41	4.44	32.47	2.28	18.12	1.84	19.26	1.76
GO-EDTA	33.74	2.99	24.19	2.23	42.07	3.31	41.43	3.4
GO-CS	46.32	2.316	32.51	3.9	21.17	2.21	29.11	3.2

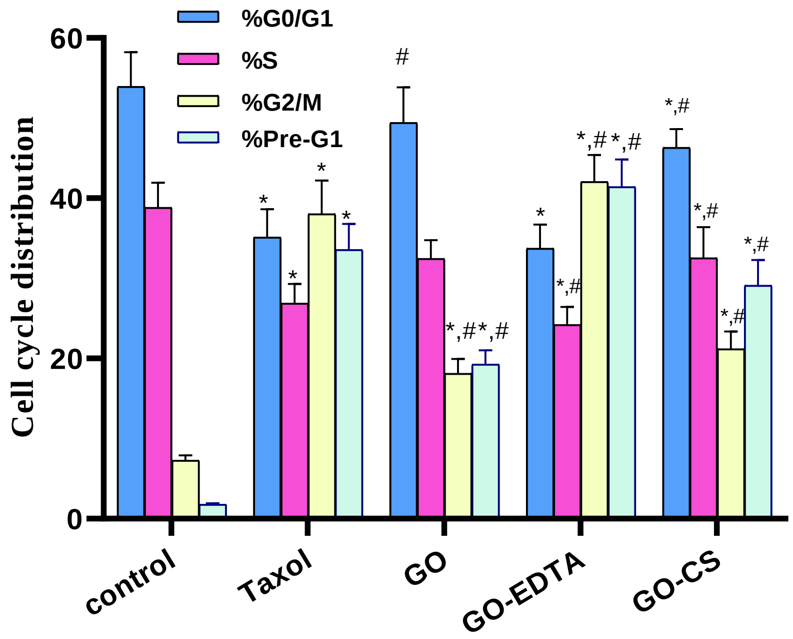

%G0/G1
%S
%G2/M
%Pre-G1
Cell cycle distribution
60
40
20
0
control
Taxol
GO
GO-EDTA
GO-CS
*
#
*,#
